# A New Oriental Species of *Behningia* Lestage (Ephemeroptera: Behningiidae)

**DOI:** 10.1673/031.006.4701

**Published:** 2006-12-31

**Authors:** W. P. McCafferty, Luke M. Jacobus

**Affiliations:** Department of Entomology, Purdue University, West Lafayette, IN 47906

**Keywords:** mayfly, *Behningia baei* new species, tuskless burrower, Thailand

## Abstract

A new species of primitive tuskless burrowing mayflies (Ephemeroptera: Palpotarsa: Behningiidae), *Behningia baei*, new species, is described from larvae taken in Thailand. The new species is differentiated from congeners primarily by its labial palps, labrum, and base of the mid legs. It is the first species of the genus *Behningia*, and only the second species of the family Behningiidae, to be taken from the Oriental biogeographic region. Larvae previously regarded as *B. tshernovae* Edmunds and Traver are considered to be assignable to *B. lestagei* Motas and Bacesco.

## Introduction

Larvae taken in stream surveys in Thailand in 2002 by R. W. Sites (Parnrong et al. 2002) included larvae of the tuskless primitive burrowing mayfly family Behningiidae (Suborder Furcatergalia, Infraorder Palpotarsa) (McCafferty 2004). These larvae proved to represent a new species of the Old World genus *Behningia* (see Edmunds and Traver 1959) that is described herein. The very striking and unusual larvae were taken in lotic sand substrate, where behningiids typically live as interstitial predators, e.g., see overview of the closely related North American *Dolania* Edmunds and Traver by McCafferty (2005).

## Taxonomy

### *Behningia baei* McCafferty and Jacobus, new species

#### Larva

**Figures 1–7.**
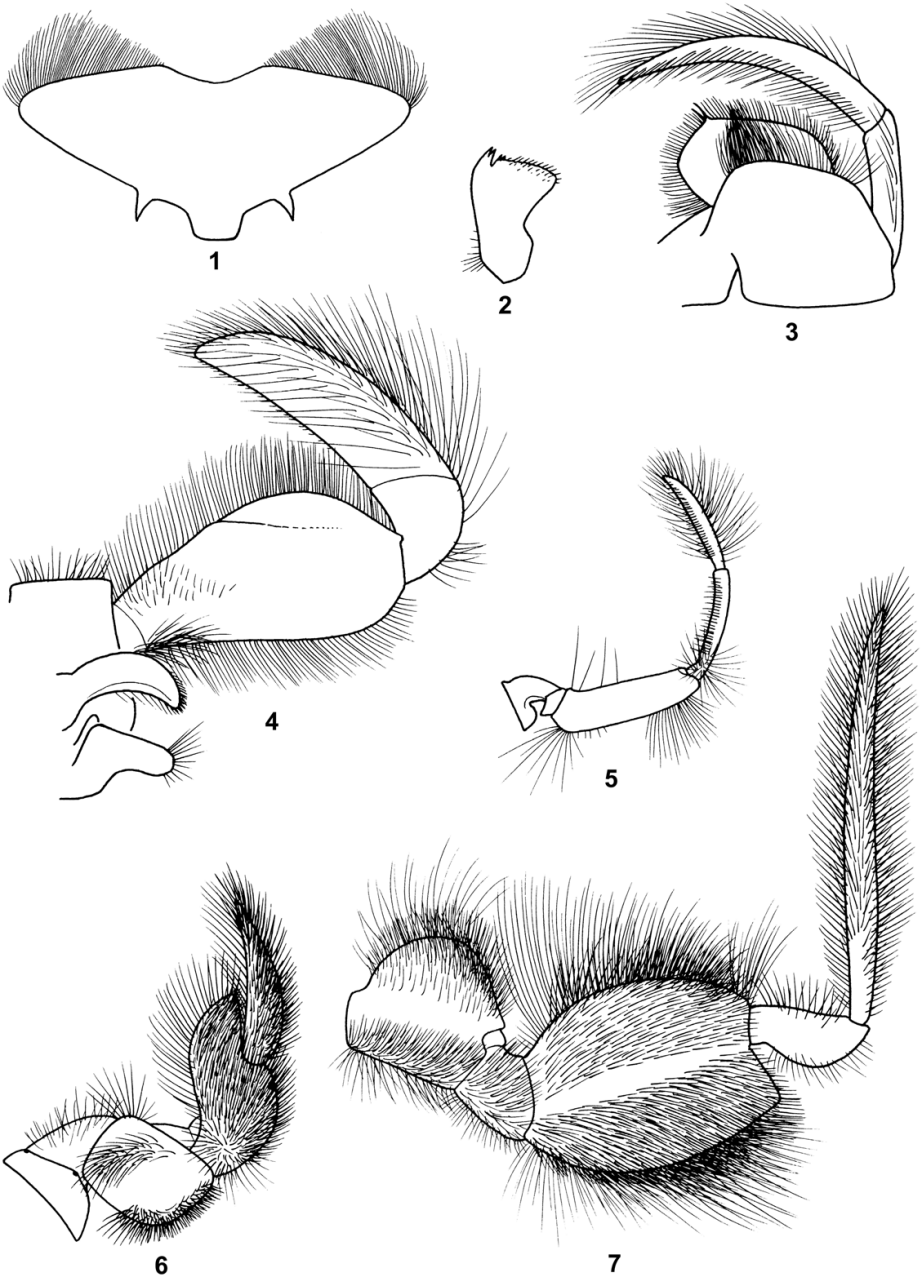
*Behningia baei*, larva (all structures drawn to scale), 1, Labrum. 2, Mandible. 3, Maxilla. 4, Labium. 5, Foreleg. 6, Midleg. 7, Hindleg.

Mature body length 13.0 mm; caudal filaments 6.5 mm. General coloration ventrally light, dorsally medium smoke gray with few light markings. Mouth parts heavily setaceous, more so than shown in Figs. 1–4. Labrum (Figure 1) broad, with broad medioanterior emargination 0.27X labral width. Mandible as in Figure 2. Maxilla as in Figure 3; outer margin of enlarged palp segment 1 straight for entire length. Labium (Figure 4) with narrow, slightly curved glossa; palp segment 1 with straight margins from base, without concavities along either margin, with width gradually increasing to greatest width at about three-fourths length from base and then tapering distally, with greatest width 0.42X length; palp segment 2 relatively short, 0.34X length of segment 3. Fore leg as in Figure 5. Mid leg as in Figure 6; trochanter longer than coxa. Hind leg as in Figure 7. Abdominal terga with paired submedian light markings, progressively more developed in posterior segments; tergum 9 with white, irregularly margined anterior area; tergum 10 mostly light with diffuse gray markings. Caudal filaments cream, unmarked.

*Material examined*: Holotype, late instar larva, Thailand, Phitsanoluk Prov., Amphur Chat Trakan, Klong Namkub at Ban Coke Huan, 17°17′N, 100°38≈E, L- 284, 10, March, 2002, Sites, Vitheepradit, Kirawanich (deposited in the Purdue Entomological Research Collection, West Lafayette, Indiana, U.S.A.). Other material: six middle instar larvae with same collecting data as holotype; three with same depostion as holotype, and three deposited in the Wilbur Enns Entomology Museum, University of Missouri, Columbia, Missouri, U.S.A. For comparative purposes we also examined larval material at Purdue of other *Behningia* species larvae as follows: *B. lestagei* Motas and Bacesco, Poland, Warta, Ostrowska, 11-VI-1960, 12-VI-1904; and *B.* sp. A, Poland, Warta, Kuczki, 22-VI-1958.

*Etymology*: The species is named for Professor Jae Bae, our esteemed ephemeropterist colleague from Seoul, Korea.

## Discussion

*Behningia baei* represents the only known species of the genus *Behningia* outside the Palearctic, and only the second species of Behningiidae known from the Orient, where *Protobehningia merga* Peters and Gillies is also known from Thailand. The cladistic evidence is compelling that *Behningia* and the Nearctic genus *Dolania* are sister genera and represent a clade opposite the more plesiotypic *Protobehningia* Tshernova (Palearctic/Oriental) (Peters and Gillies 1991). The former pair share such specialized characteristics as the loss of the tarsal claw and reduced hind tibiae in the larvae and fusion of the basal forceps segment in the adults.

Comparisons of *B. baei* with other species of *Behningia* are based on material we have of *B. lestagei* and a presumably unnamed species very similar or equivalent to *B. ulmeri* Lestage, which we refer to as *B.* sp. A, in addition to published data currently associated with *Behningia* larvae. As further shown below, no essential basis has been found for recognizing the larvae previously associated with *B. tshernovae* as being different than the larvae of *B. lestagei*. The type of *B. tshernovae* is based on adults figured by Tshernova (1938), and the association of larvae by Edmunds and Traver (1959) was represented tentatively only by Figure 23 (Edmunds and Traver 1959) of the labial palp of a questionable larva incompletely treated by Tshernova (1952). We here consider those larvae formerly associated with *B. tshernovae* to be *B. lestagei*, and the larvae of *B. tshernovae*, if a valid species, to be undescribed.

Edmunds and Traver (1959) diagnosed the species of *Behningia* in the larval stage by way of the shape of the enlarged palp segment 1 and the relative length of palp segment 2 of the highly specialized labium. *Behningia baei* can also be diagnosed using these same characters. First, *B. baei* (Figure 4) differs from *B. ulmeri* (Figure se in Ulmer 1924), but is similar to *B. lestagei* (Figs. 19 and 23 in Edmunds and Traver 1959) and *B.* sp. A by having a relatively short palp segment 2. Second, *B. baei* (Figure 4) differs from *B. ulmeri* (Figure 3e in Ulmer 1924) and *B.* sp. A, but is similar to *B. lestagei* (Figs. 19 and 23 in Edmunds and Traver 1959) by having a relatively more broadened palp segment 1. The shape of palp segment 1 of *B. baei* (Fig. 4) differs significantly from that of *B. lestagei*, in that it lacks any concavity along the margins. *Behningia lestagei* (Figs. 19 and 23 in Edmunds and Traver 1959) has a distinct concavity along the outer margin of palp segment 1 at or nearly at the midway point from the base, and it also has a concavity in much of the basal half of the inner margin. The shapes of palp segment 1 in *B. lestagei* (Fig. 19 in Edmunds and Traver 1959) and larvae previously assigned to *B. tshervnovae* (Fig 23 in Edmunds and Traver 1959) appear essentially the same; and they differ ostensibly only in that the width of palp segment 1 appears slightly broader based on the figure comparison.

We have also found the labrum to be of some use in distinguishing *B. baei, B. lestagei, B. ulmeri*, and *B.* sp. A. In *B. baei* (Figure 1), the labrum is broadly emarginated and quite similar in that respect to *B. ulmeri* (Figure 3d in Ulmer 1924), where the medial emargination is broadly V shaped but somewhat narrower than that of *B. baei* (slightly less that .25X the width of the labrum). In *B.* sp. A, the emargination is somewhat deeper and more narrowly V shaped than in *B. ulmeri*. In *B. lestagei* (Fig. 16 in Edmunds and Traver 1950), the emargination is shallow, narrower, and more U shaped than in *B. ulmeri*. One other feature that may be of some limited use in diagnosing *B. baei* is the size of the mid trochanter relative to the mid coxa. In *B. baei* (Figure 6), *B. ulmeri* (Figure 5b in Ulmer 1924), and *B.* sp. A, the trochanter is considerably longer than the coxa; whereas, in *B. lestagei*, the trochanter is highly reduced (Fig. 14 in Edmunds and Traver 1959).
